# Community-level influences on women’s experience of intimate partner violence and modern contraceptive use in Nigeria: a multilevel analysis of nationally representative survey

**DOI:** 10.12688/aasopenres.13247.2

**Published:** 2021-11-08

**Authors:** Sunday A. Adedini, Ololade Grace Adewole, Funmilola F. Oyinlola, Olufunke Fayehun

**Affiliations:** 1 Demography and Social Statistics Department, Federal University Oye-Ekiti, Oye-Ekiti, Ekiti State, 371106, Nigeria; 2 Demography and Population Studies Programme, Schools of Social Science and Public Health, University of the Witwatersrand, Johannesburg, Gauteng, 2050, South Africa; 3 NACETEM, Obafemi Awolowo University, Ile-Ife, Osun, 220005, Nigeria; 4 Demography and Social Statistics Department, Obafemi Awolowo University, Ile-Ife, Osun, 220005, Nigeria; 5 Department of Sociology, University of Ibadan, Ibadan, Oyo State, 200284, Nigeria

**Keywords:** Contraceptives use, intimate partner violence, community contexts, Nigeria

## Abstract

***Background***: Modern contraceptives (MC) are important strategies for reducing unwanted pregnancies, unsafe abortion and maternal mortality, but MC remains low at 18% in Nigeria. Similarly, while there is increasing prevalence of intimate partner violence (IPV) in Nigeria, its effects on contraceptive use remain unclear. This study examined the influence of IPV on MC use, while adjusting for individual- and community-level confounders.

***Methods***: The study utilized 2018 Nigeria Demographic and Health Survey data. We performed multilevel binary logistic regression analysis on 24,973 married women aged 15–49 49 (nested within 1,400 communities), who were sexually active and were not pregnant at the time of the survey.

***Results***: Findings show that use of MC was higher among married women who reported experience of IPV than those without IPV exposure. After adjusting for individual-level and contextual factors, the odds of using MC was significantly higher among women who experienced any form of IPV (OR: 1.61, 95% CI: 1.17-2.21, p<0.005) compared to those who reported no IPV experience. Around one-quarter of the total variance in contraceptive use with respect to the different types of IPV could be explained at the community level.

***Conclusion***: The study provides empirical evidence that there is signiﬁcant community effect on IPV exposure and women’s contraceptive uptake. Attention must therefore be given to the context-specific social and gender norms that affect women’s sexual and reproductive health in Nigeria.

## Introduction

Modern contraceptive is one of the most important means of reducing unwanted pregnancies, abortions and maternal mortality. It also serves as a good strategy for women to achieve their fertility intentions and improve their socio-economic status ^1, 2^. Despite the numerous benefits of contraception, recent data shows that modern contraceptive prevalence rate (mCPR) remains low at 18% in Nigeria ^3^ Nigeria’s mCPR is by far lower than that of many sub-Saharan African countries like Malawi, Rwanda, and South Africa, which have mCPR of 50% or higher ^4^. A recent analysis that used three consecutive Nigeria DHS datasets revealed that in 2003 (91.8%), 2008 (90.6%) and 2013 (88.6%), most women of reproductive ages were not using contraceptives, and unmet needs for family planning fluctuate between 14% and 20% in the past 15 years ^5^. As a result of this, total fertility rate (TFR) remains high at 5.5 children per woman, with a sub-national TFR which is as high as 7.7 in some regions of the country ^3^.

The decision to use modern contraceptives by married women in developing countries, particularly those in an abusive relationship is a complex ^6, 7^ one. For instance, payment of bride price reinforces the belief that a woman is required to provide sexual satisfaction for her partner and also bear children. On the other hand, literature has established that men often express anxiety and belief that link sexual infidelity to the practice of modern contraception among women ^8^. Such beliefs can serve as a precursor for gender-based violence. There is some significant evidence in the literature that family planning and contraceptive use may exacerbate tension in gender relations ^9, 10^. In many societies where culture and religion are predominantly adhered to, women are subjected to seeking spousal or partner consents on their sexual and reproductive health decision. This has resulted to patriarchal control which is one of the important underlying barriers for family planning program in developing countries ^11, 12^. The decision to use contraceptives have been found to be better where couples agree ^13– 16^ Further, the consensus on decision to use contraceptives might not be easily reached in cases where fertility desire of either the husband or wife are yet to be met, this could lead to disagreement or even intimate partner violence.

Further, prior research shows that women who are exposed to sexual and emotional violence are most likely to be denied access to emergency contraceptives ^17, 18^. It is very important to emphasize that intimate partner violence worsens women’s decision-making capacity for herself ^19^ and her children particularly due to lack of control over socioeconomic resources ^20^. Although many studies have explored the influence of intimate partner violence on contraceptive use, available evidence on the relationship between the two phenomena is mixed. For instance, Pallitto & Campbell ^21^, found that women who experienced IPV were less likely to negotiate contraceptive use and safer sex than those without such experience. In addition, a study by 22 and 23 established IPV as a barrier to contraceptive use, while in contrast ^24^, found that women who experienced IPV had higher likelihood of using contraceptives than those who had no exposure to IPV. Moreover, there is little systematic evidence on how community structure affects IPV and modern contraceptive uptake; whereas community-level factors are critical to women’s reproductive health ^25^ This is because certain gendered norms and traditional cultural values tend to inhibit usage of modern contraception ^26^. Besides, IPV is contextually and socially derived from entrenched gender norm and traditional cultural values ^27, 28^. Thus, the key research question for this study was: ‘to what extent does married women’s experience of IPV (net of individual- and community-level characteristics) inﬂuence contraceptive use in Nigeria?

## Some theoretical perspectives on IPV and contraceptive use

The study is guided by the theory of gender and power (TGP). Postulated by Connell ^29^ TGP hypothesizes that sexual inequality between men and women articulates gender-based inequities within the structure and institutions of the societies. It also assumed that though gender inequity may be external to the individual, it plays a role in individual action that is beyond his/her control. The theory described some risk factors linked to male dominance and power imbalances that adversely affect women’s health by identifying three major structures. The three major structures, which deal with constraints experienced by women in the society, are sexual division of labour, sexual division of power, and structure of cathexis. The sexual division of labour is on the unequal allocation of occupations and positions for women when compared to their men counterparts. Consequently, the economic imbalance is created and women are forced to depend on men for financial assistance since several compensated responsibilities are structurally and institutionally designed in favour of men at the detriment of the women.

The sexual division of power is on the social mechanisms put in place by men to exercise control and influence over women. Men dominate critical societal issues, depending on the matters to which authority and control are to be exercised ^30^. Relative to women’s power in the household, their powers are constrained by the social mechanisms of patriarchy to disempower and subjugate them, which may have implications on the abuse of women socially and sexually. While the sexual divisions of labour, and power seem to focus on economic and decision-making process, the structure of cathexis tends to address issues around affective attachments and social norms. It is mainly concerned with the dictate of appropriate sexual behaviour for women as characterized by their emotions and sexual attachments with men. In this regard, women are constrained by the social norms that shape their sexual behaviour in such a manner that deny them from exercising their sexual and reproductive rights ^30^ This further suggests that women who are more receptive to the social norms are more likely to experience IPV

In applying this theory, women are to subject themselves to the desire of men in any matter related to modern contraceptive method because of low economic power to make a choice. In a situation where men are not supportive of women’s use of contraceptive, there may be a likelihood of the risk of IPV and covert use or none use of contraceptives.

Also, the sexual division of power between men and women could be a risk factor for IPV and contraceptive use. This is where power imbalances are often used to describe how partner abuse emerge ^30, 31^. This is because sexual division of power accentuates authority and influence vested in men. Consequently, women are expected to submit to men’s authority and control on sexual matters. The culturally-laden social norms on intimate partner relationship reinforces women’s perception in traditional societies that denying husband’s sex is culturally and religiously unacceptable ^32^. This could be one of the justifications for the prevalence of marital rape in most sub-Saharan African countries which are not always reported for prosecution ^33, 34^.

While the first two components of Connell’s (1987) TGP – *structure of division of labour and power* - seem to illustrate the risk factors of IPV as applied to this study, the structure of cathexis tends to focus more on the social norms that may serve as constraints to the use of contraceptive among women. These constraints may include the age of partners in marital relationship, emotions, belief systems about the use of contraception, fertility intentions of both partners, family influence and availability of family planning methods. For example, there are evidences in some African countries that women who initiate the use of modern contraceptive may be accused of promiscuity by their spouse ^35, 36^. This may mostly restrain most women from making contraceptive use decision or else seek the approval of their spouses. It is covertly used where no approval is given ^37^, and such a woman may stand the risk of IPV. This implies that both structures of TGP are interwoven in the explanations of IPV and contraceptive use. The occurrence of imbalances in both structures of TGP could be critical risk factors of IPV, and consequently, none use or covert use of contraception. These have policy implications for gender equality and empowerment. 

## Methods

The study employed secondary data extracted from the individual recode file of 2018 Nigeria Demographic and Health Survey (NDHS). The sampling frame for the survey was the list of enumeration areas (EAs) prepared for the 2006 Population and Housing Census of Nigeria, provided by the National Population Commission. The sample was selected using a stratified two-stage cluster design consisting of 1400 clusters with enumeration areas (EAs) as the sampling units for the first stage. A sample of 42,000 households was selected for the survey, with 99.3% and 99.5% response rate in urban and rural areas, respectively. The unit of analysis for the present study was currently married women aged 15–49 years; however, those who had never had sex or were pregnant at the time of the survey were excluded from the analysis. A weighted sample of 24973 women aged 15–49 (nested within 1,400 communities) was the analytic sample for the study.

### Variables measurements

***Outcome variable.*** The outcome variable for this study was contraceptive use. This was defined as current use of modern method of contraception (at the time of the survey) by married women aged 15–49 years. The variable was categorized into two: (i) currently using at least one modern method of contraception, categorized as ‘1’, and (ii) not using any modern method, categorized as ‘0’.

***Explanatory variables.*** In terms of exposure, our key explanatory variable was intimate partner violence (IPV). IPV was defined as ever-experience of domestic violence (in terms of physical, sexual and emotional violence) from intimate partner or spouse. This variable was recorded in the domestic violence module of 2018 NDHS, using different dimensions. We generated IPV using the three different dimensions of violence captured in the domestic violence module of the survey as shown below:

(A). A woman is said to have experienced physical violence if there was a “yes” response to any of the seven questions below:

*Does (did) your (last) husband/partner ever do any of the following things to you?*

*i) Push you, shake you, or throw something at you? ii) Slap you? iii) Twist your arm or pull your hair? iv) Punch you with his fist or with something that could hurt you? v) Kick you, drag you, or beat you up? vi) Try to choke you or burn you on purpose? vii) Threaten or attack you with a knife, gun, or any other weapon?*

(B). There is evidence of sexual violence if a woman responded “yes” to any of the three questions below:

*Does (did) your (last) husband/partner ever do any of the following things to you:*

*i)*  *Ever been physically forced into unwanted sex by husband/partner*
*ii)*  *Ever forced into other unwanted sexual acts by husband/partner*
*iii)*  *Ever been physically forced to perform sexual acts respondent didn’t want to*

(C). A woman is said to have experienced emotional violence if there is at least one “yes” answer to any of the following three questions:

*Does (did) your (last) husband/partner ever do any of the following things to you?*

*i) Say or do something to humiliate you in front of others? ii) Threaten to hurt or harm you or someone close to you? iii) Insult you or make you feel bad about yourself?*

Physical violence was examined using items Ai-Avii (Cronbach’s alpha=0.75); sexual violence was assessed by items Bi-Biii (Cronbach’s alpha=0.74); while emotional violence was examined based on items Ci-Ciii (Cronbach’s alpha=0.64). Ever-experience any form of IPV was measured by all the 13 items (Cronbach’s alpha=0.81).

Other explanatory variables controlled for in the analysis were selected based on the guidance from the reviewed literature and theoretical underpinning for the study. The variables included individual level characteristics— current age (15–24; 24–34; and 35 above), level of education (no education; primary, secondary, and higher education), children ever born (<5 and 5+), religion (Islam, Christianity, and others); ethnicity (Hausa, Igbo, Yoruba and others); age at marriage (<18, 18–24 and 25+); occupation (formal, informal and manual labour) and wealth status (poorest, poorer, rich, richer and richest). The selected community level variables include region of residence (North Central, North East, North West, South-South and South West); place of residence (rural/urban residence), ethnic diversity (the extent of diversity in the community where respondents live in terms of ethnic composition, categorized as homogeneous, mixed and heterogeneous); community level of education (proportion of women who had at least secondary education in the community, grouped as low, medium and high); community media access (proportion of women who had access to newspaper/magazine, radio and television — low, medium and high); and community poverty level (proportion of women with low income level in the community, categorized as low, medium and high). Most of the level two variables were generated by aggregating the individual-level variables at the level of primary sampling units (PSUs).

### Statistical analysis

The study employed descriptive and inferential statistics in data analysis, including frequency and percentage distribution (univariate analysis); cross-tabulation and Chi-square test (bivariate analysis); and multilevel binary logistic regression (multivariable analysis). Leveraging on the hierarchical nature of the NDHS data, we – specified a 2-level multilevel modelling (individual- and contextual-levels) for the binary outcome (use or non-use of modern contraception). Crude models examined the effect of IPV type on the use of modern contraceptives, while the adjusted models incorporated the individual (level 1) and contextual (level 2) variables. Influence of IPV was examined on modern contraceptive use (the outcome variable). We have 4 different sub-samples for the explanatory variable – (i) physical IPV sub-sample (ii) sexual IPV sub-sample (iii) emotional IPV sub-sample and (iv) respondents who reported any IPV. Different models focus on each of these 4 sub-samples.

The fixed effects (that is, measures of association) were expressed as odds ratios (ORs) with 95% confidence intervals (95% CIs). The random effects which were regarded as measures of variations in intimate partner violence and contraceptive use across communities were expressed as intra-class correlation (ICC) (or variance partition coefficient (VPC)), and proportional change in variance (PCV). To determine the goodness of fit of the consecutive models, regression diagnostic was done using Akaike Information Criteria (AIC). Evidence suggests that the lower value of AIC indicates a better fit (Boco 2010).All analysis was done using Stata (version 13.0).

## Results

### Prevalence of contraceptive use by IPV types and selected characteristics

Table 1 presents the percentage distribution of individual level characteristics by contraceptive use and according to IPV types. The table shows that 35.6% of women reported experience of any form of violence, 30.9% experienced emotional violence, 18.8% had suffered physical violence and 6.3% reported experience of sexual violence. Relative to other married women, use of modern contraceptives was higher among women who had less than 5 children (14.4%), were aged 25–34 years (15.9%), were Christian (22.3%), were Yorubas (27.5%), married between 18–24 years (20.0%), had higher education (25.7), had formal employment (21.1) and were from the richest households (24.9%). The use of modern contraceptive was also higher among married women who reported experience of IPV than those who did not have IPV exposure. The percentage was 19.1% for those who had experienced any form of violence; and 18.7%, 20.4% and 18.0% among those who reported emotional violence, physical violence, and sexual violence, respectively.

**Table 1.** Percentage distribution of individual-level characteristics of the study population by contraceptive use (NDHS 2018).

Variables	Contraceptive use				
	Yes N (%)	No N (%)	Total N (%)	P Value	χ²
**Physical violence**					
No physical violence	929 (15.8)	4937 (84.2)	5866 (81.2)	0.000	16.2614
Experienced physical violence	276 (20.4)	1079(79.6)	1355(18.8)		
**Sexual violence**					
No sexual violence	1123 (16.6)	5645 (83.4)	6767 (93.7)	0.416	0.6604
Experienced sexual violence	82 (18.0)	372 (82.0)	454 (6.3)		
**Emotional violence**					
No emotional violence	787 (15.8)	4201 (84.2)	4988 (69.1)	0.002	9.5986
Experienced emotional violence	418 (18.7)	1815 (81.3)	2233 (30.9)		
**Any form of violence**					
None of the three forms	717 (15.4)	3936 (84.6)	4652 (64.4)	0.000	15.3138
Experienced any of the three forms	488 (19.0)	2081 (81.0)	2569 (35.6)		
**Children ever born**					
<5 children	2160 (14.4)	12825 (85.6)	14985 (60.0)	0.031	4.6729
5+ children	1343 (13.4)	8646 (86.6)	9988 (40.0)		
**Religion**					
Christianity	1771 (22.3)	6160 (77.7)	7931 (31.8)	0.000	750.0
Islam	1342 (9.2)	13299 (90.8)	14641 (58.6)		
Others	390 (16.2)	2012 (83.8)	2402 (9.6)		
**Current age**					
15–24	402 (8.3)	4467 (91.7)	4870 (19.5)	0.000	169.0925
25–34	1509 (15.9)	8005 (84.1)	9515 (38.1)		
35 and above	1591 (15.0)	8998 (85.0)	10588 (42.4)		
**Ethnicity**					
Hausa	977 (7.9)	11394 (92.1)	12371 (49.6)	0.000	1.0e+03
Igbo	571 (17.3)	2742 (82.8)	3313 (13.3)		
Yoruba	1030 (27.5)	2720 (72.5)	3750 (15.0)		
Others	922 (16.7)	4600 (83.3)	5522 (22.1)		
**Educational Level**					
No education	556 (5.1)	10357 (94.9)	10913 (43.7)	0.000	1.1e+03
Primary	646 (16.0)	3406 (84.0)	4053 (16.2)		
Secondary	1667 (22.1)	5872 (77.9)	7539 (30.2)		
Higher	633 (25.7)	1835 (74.3)	2468 (9.9)		
**Age at first marriage**					
<18	1206 (9.3)	11710 (90.7)	12916 (51.7)	0.000	510.8156
18–24	1726 (20.0)	6937 (80.0)	8670 (34.7)		
25+	563 (16.6)	2824 (83.4)	3386 (13.6)		
**Wealth index**					
Poorest	221 (4.4)	4797 (95.6)	5018 (20.1)	0.000	1.2e+03
Poorer	390 (7.5)	4822 (92.5)	5212 (20.9)		
Middle	628 (13.2)	4136 (86.8)	4763 (19.1)		
Richer	1005 (20.4)	3918 (79.6)	4923 (19.7)		
Richest	1259 (24.9)	3798 (75.1)	5027 (20.3)		
**Occupation**					
No employment					
Formal	712 (21.1)	2667 (78.9)	3379 (17.9)	0.000	88.7756
Informal	2123 (14.6)	12473 (85.5)	14596 (77.5)		
Manual labour	149 (17.1)	723 (82.9)	872 (4.6)		

Percentage distribution of community-level characteristics of the study population by contraceptive use was presented in Table 2. At the community level, contraceptive use was highest among women residing in the South-West region (26.7%). It was also significantly (p<0.005) higher in communities with a high proportion of educated women (23.1%), urban areas (20.8), rich communities (20.0%), heterogeneous communities (19.9%), and communities with high media exposure (19.2%).

**Table 2.** Percentage distribution of community-level characteristics of the study population by contraceptive use (NDHS 2018).

Variables	Contraceptive use				
	Yes N (%)	No N (%)	Total N (%)	P Value	χ²
**Regions**					
North Central	563 (16.2)	2912 (83.8)	3475 (13.9)	0.000	974.8783
North East	379 (9.4)	3673 (90.6)	4052 (16.2)		
North West	613 (7.5)	7562 (92.5)	8175 (32.7)		
South East	373 (14.7)	2162 (85.3)	2535 (10.2)		
South South	438 (17.6)	2051 (82.4)	2488 (10.0)		
South West	1137 (26.7)	3111 (73.2)	4247 (17.0)		
**Place of residence**					
Urban	2151 (20.8)	8194 (79.2)	10345 (41.4)	0.000	670.3257
Rural	1352 (9.2)	13277 (90.8)	14628 (58.6)		
**Ethnic diversity**					
Homogenous	733 (7.6)	8873 (92.4)	9606 (38.5)	0.000	576.4625
Mixed	1212 (16.1)	6330 (83.9)	7541 (30.2)		
Heterogeneous	1558 (19.9)	6268 (80.1)	7825 (31.3)		
**Community poverty**					
Low	1946 (20.0)	7757 (80.0)	9703 (38.9)	0.000	654.9937
Medium	1043 (14.0)	6399 (86.0)	7442 (29.8)		
High	513 (6.6)	7314 (93.4)	7828 (31.3)		
**Community level of** **education**					
Low	480 (4.6)	9856 (95.4)	10336 (41.4)	0.000	1.4e+03
Middle	1140 (17.6)	5351 (82.4)	6491 (26.0)		
High	1882 (23.1)	6263 (76.9)	8145 (32.6)		
**Community media access**					
Low	752 (11.2)	5958 (88.8)	6710 (26.9)	0.000	341.4524
Middle	934 (10.6)	7879 (89.4)	8814 (26.9)		
High	1816 (19.2)	7633 (80.8)	9449 (37.8)		

### Multivariable analysis of relationship between contraceptive use, IPV types and selected individual- and community-level characteristics

Table 3 shows the fixed and random effects for the crude and adjusted ORs exploring the relationship between contraceptive use and different IPV types (net of selected individual- and community-level characteristics). In the crude models, all the IPV types (with the exception of sexual IPV) were significantly associated with women’s use of contraception (p<0.005). Among married women, the odds of using modern contraceptive was 1.79 times higher among those who experienced physical violence, and 1.44 times higher among those who experienced emotional violence than those who reported otherwise. Irrespective of the type of violence, the likelihood of using contraceptive was higher among married women who had experienced any form of IPV than those who had no experience of IPV.

**Table 3.** Results from multilevel logistic regression showing the influence of intimate partner violence on contraceptive use among married women in Nigeria (NDHS 2018).

Characteristics	OR (95% CI)		OR (95% CI)		OR (95% CI)		OR (95% CI)	OR (95% CI)
	Crude	Adjusted	Crude	Adjusted	Crude	Adjusted	Crude	Adjusted
**Physical IPV**								
Yes (No = ref)	1.79* (1.21-2.63)	1.34 (0.96-1.87)						
**Sexual IPV**								
Yes (No = ref)			1.34 (0.79-2.26)	1.31 (0.77-2.22)				
**Emotional IPV**								
Yes (No = ref)					1.44* (1.05-1.99)	1.50 (0.53-1.67)		
**Any of the three forms of IPV**								
Yes (No = ref)							1.64* (1.18-2.30)	1.61* (1.17-2.21)
**Children ever born**								
5+ children (<5 children=ref)		2.88* (1.79-4.63)		2.89* (1.80-4.65)		2.90* (1.80-4.62)		2.89* (1.80-4.62)
**Religion**								
Islam (Christianity=ref)		0.34* (0.20-0.57)		0.33* (0.19-0.55)		0.34* (1.80-4.62)		2.89* (1.80-4.62)
Others		1.12 (0.73-1.73)		1.12 (0.73-1.73)		1.12 (0.72-1.72)		1.11 (0.72-1.72)
**Current age**								
25–34 (15-24=ref)		1.25 (0.79-2.00)		1.26 (0.79-2.01)		1.23 (0.77-1.96)		1.23 (0.77-1.96)
35 and above		0.92 (0.54-1.55)		0.92 (0.55-1.55)		0.90 (0.53-1.53)		0.90 (0.53-1.53)
**Ethnicity**								
Igbo (Hausa=ref)		2.20* (1.01-4.79)		2.20* (1.01-4.79)		2.27* (1.02-4.89)		2.24* (1.02-4.90)
Yoruba		3.75* (1.69-8.30)		3.81* (1.71-8.46)		3.82* (1.71-8.42)		3.80* (1.71-8.42)
Others		2.01* (1.14-3.55)		2.02* (1.14-3.57)		2.04* (1.14-3.57)		2.02* (1.14-3.57)
**Educational Level**								
Primary (No education=ref)		2.19* (1.28-3.76)		2.18* (1.27-3.73)		2.18* (1.02-4.89)		2.18* (1.27-3.75)
Secondary		4.44* (2.28-8.68)		4.42* (2.27-8.63)		4.46* (2.27-8.57)		4.42* (2.28-8.58)
Higher		5.37* (2.38-2.12)		5.25* (2.33-11.79)		5.42* (2.4-12.2)		5.44* (2.41-2.3)
**Age at marriage**								
18–24 (<18=ref)		0.94 (0.68-1.30)		0.94 (0.68-1.30)		0.94 (0.68-1.30)		0.94 (0.68-1.30)
25+		0.35* (0.20-0.62)		0.34* (0.19-0.61)		0.35* (0.20-0.63)		0.35* (0.20-0.62)
**Wealth index**								
Poorer (Poorest=ref)		1.01 (0.56-1.83)		1.01 (0.56-1.83)		1.01* (0.56-1.83)		1.01 (0.56-1.83)
Middle		1.46 (0.79-2.75)		1.49 (0.79-2.73)		1.48 (0.80-2.79)		1.49 (0.80-2.79)
Richer		1.84 (0.93-3.70)		1.89 (0.92-3.68)		1.89 (0.94-3.79)		1.89 (0.94-3.79)
Richest		2.24* (1.04-4.95)		2.33* (1.03-4.88)		2.29* (1.06-5.10)		2.33* (1.06-5.10)
**Occupation**								
Informal (Formal=ref)		1.44 (0.99-2.121		1.44 (0.99-2.11)		1.45 (0.99-2.1)		1.46 (0.99-2.13)
Manual labour		1.50 (0.78-2.88)		1.51 (0.78-2.89)		1.51 (0.78-2.91)		1.51 (0.78-2.91)
**Regions**								
North East (North Central)		0.92 (0.52-1.62)		0.90 (0.51-1.60)		0.89 (0.50-1.59)		0.90 (0.50-1.59)
North West		0.96 (0.52-1.81)		0.95 (0.50-1.77)		0.96 (0.52-1.86)		0.98 (0.52-1.85)
South East		0.17* (0.07-0.44)		0.17* (0.07-0.45)		0.17* (0.07-1.86)		0.17* (0.07-0.45)
South South		0.35* (0.19-0.66		0.35* (0.19-0.66)		0.36* (0.19-0.67)		0.36* (0.19-0.67)
South West		0.87 (0.48-1.58)		0.85 (0.47-1.54)		0.94 (0.52-1.74)		0.96 (0.52-1.75)
**Place of residence**								
Rural (Urban=ref)		0.67* (0.46-0.98)		0.66* (0.46-0.97)		0.67* (0.46-0.98)		0.67* (0.46-0.98)
**Ethnic diversity**								
Mixed (Homogenous=ref)		0.85 (0.53-1.39)		0.86 (0.53-1.39)		0.84 (0.52-1.37)		0.84 (0.52-1.37)
Heterogeneous		0.84 (0.48-1.49)		0.85 (0.48-1.51)		0.83 (0.47-1.47)		0.83 (0.47-1.47)
**Community poverty**								
Medium (Low=ref)		0.90 (0.56-1.46)		0.91 (0.56-1.47)		0.89 (0.47-1.47)		0.88 (0.55-1.43)
High		1.41 (0.71-2.77)		1.0 (0.71-2.76)		1.40 (0.72-2.82)		1.42 (0.72-2.81)
**Community level of education**							
Middle		2.12* (1.23-3.65)		2.13* (1.23-3.68)		2.10* (1.23-3.67)		2.13* (1.23-3.67)
High		3.24* (1.56-6.73)		3.26* (1.57-6.77)		3.21 (1.55-6.67)		3.22* (1.55-6.67)
**Community media access**								
Middle (Low=ref)		0.95 (0.62-.46)		0.95 (0.62-1.45)		0.96 (0.62-1.46)		0.95 (0.62-1.47)
High		0.85 (0.50-1.45)		0.85 (0.50-1.46)		0.87 (0.50-1.46)		0.86 (0.50-1.46)
**Random effects**		**Physical IPV sub-** **sample**		**Sexual IPV sub-sample**		**Emotional IPV sub-** **sample**		**Any IPV ** **sub-sample**	
**Community-level**								
Variance (SE)	4.13 (1.86)	1.09 (0.45)	4.36 (1.93)	1.11 (0.45)	4.32 (1.92)	1.10 (0.44)	4.28 (1.91)	1.11 (0.44)
VPC=ICC (%)	55.7	24.9	57.0	25.2	56.8	25.1	56.5.	25.2
Explained variation (PCV) %	Reference	73.6	Reference	74.5	Reference	74.5	Reference	74.1
**Individual-level**								
Variance (SE)	7.29 (4.18)	5.79 (2.79)	7.63 (4.15)	5.81 (2.78)	7.54 (4.16)	5.85 (2.76)	7.48 (4.17)	5.88 (2.75)
Explained variation (PCV) %	Reference	20.6	Reference	23.9	Reference	22.4	Reference	21.4
Log likelihood	-3047.89	-2251.11	-3053.59	-2256.20	-3051.09	-2253.03	-3048.09	-2251.36
**Model fit statistics**								
AIC	6103.79	4584.23	6115.18	4586.39	6110.18	4580.05	6104.18	4576.72
BIC	6131.30	4828.29	6142.67	4830.46	6137.67	4824.12	6131.67	4820.78

After adjusting for individual-level and contextual factors, the odds of using modern contraceptives, though slightly reduced, remained significantly higher among women who experienced any form of IPV (OR: 1.61, 95% CI: 1.17-2.21, p<0.005) compared to those who reported no IPV experience. However, there were no significant relationships between different types of IPV and contraceptives after adjusting for individual and community factors.

In the the adjusted models for all IPV types, there was a significant relationship between contraceptive use and a number of individual- and community-level factors. These factors are CEB, religion, ethnicity, education, age at marriage, wealth index, region, place of residence, and community level of education (p<0.005).

The models show significant variances ranging from 5.79 to 7.63 across individual level, and 1.09 to 4.36 across community level. This thus justifies the use of multilevel modelling in our analysis. The VPC (or ICC), which is the estimate of relatedness or variance in women’s use of contraceptives across communities indicates that contraceptive use was not homogenous across different contexts. The ICC for contraceptive use in the adjusted models was 24.9% for physical IPV, 25.2% for sexual IPV, 25.1% for emotional IPV, and 25.2% for any type of IPV. These findings show that around one-quarter of the total variance in contraceptive use with respect to the different types of IPV could be explained at the community level. The estimates from AICs which are the goodness of fit for the adjusted model were smaller than those of crude models, hence they present the better fits.

## Discussion

Intimate partner violence is a leading public health concern with grave consequences for women’s physical, mental, sexual and reproductive health. Our results established that more than one-third of married women experienced at least one form of IPV. This suggests an increase in IPV prevalence as compared with previous study that established 23% IPV prevalence among married women from analysis of 2013 Nigeria DHS ^27^. Interestingly, we found that women who experienced different forms of IPV reported higher uptake of contraceptives than those who experienced no IPV. Previous studies have established similar findings. A study in Democratic Republic of the Congo established a positive association between IPV and modern contraceptive use ^38^. Also, a multi-country study of 6 sub-Saharan African countries established that women who experienced IPV had significantly higher odds of using contraceptives than those who reported no experience of IPV ^24^. This result has implication for women’s fertility intention and preference. Plausible explanation for high contraceptive use among women who experienced IPV is that occurrence of gender imbalances and exposures to the risk of violence in a marital dyad may force women to resort to a covert use of contraception ^37, 39^ in order to prevent having many children in an abusive union. Covert contraception (CC), which is the practice of using a family planning method without a partner’s knowledge, is a common practice among women in SSA ^40^. Scholars have adduced that CC reflects the degree of how much autonomy a woman is able to exercise regarding her reproductive choices ^41, 42^. Further, contraceptive use could trigger violence within a marital union that has palpable gender inequality. Given that women in some parts of Nigeria typically desire smaller families than their husbands/partners ^43^, such desire may be accentuated by women’s exposure to spousal violence.

The pattern of higher likelihood of contraceptive uptake among women who experienced IPV relative to those with no exposure to IPV also accords with evidence from a few previous studies ^9, 44^, but contrasts others which found a lower likelihood of contraceptive use among women in abusive relationship than those in non-abusive union ^25, 45^. The lack of consensus in literature perhaps reflect uniqueness in societal gender norms in relation to culture of violence and reproductive health practices on contraception across different contexts ^44^.

Our second hypothesis in this study was that contexts may play a significant role in the association between IPV and contraceptive uptake because domestic violence is a product of social context ^25^. Based on this hypothesis, we explored the roles of community-level factors on the relationship between IPV and contraceptive use. To the best of our knowledge, this is the first study to investigate this. Our findings provide some evidence that there are signiﬁcant neighbourhood effects on the relationship between two measures of IPV (physical IPV and emotional IPV) and modern contraceptive use. After incorporating community-level variables into the multilevel model, the relationship between two measures of IPV (physical IPV and emotional IPV) and modern contraceptive use became statistically insignificant.

The study established significant community variances in contraceptive uptake. Our estimate of relatedness or variance in women’s use of contraceptives shows that relationship between IPV and contraceptive use is not homogenous across communities. The findings show that around one-quarter of the total variance in contraceptive use with respect to the different types of IPV could be explained at the community level. This suggests that community contextual factors play significant roles in the decision to use contraceptives among women exposed to IPV. For instance, IPV-exposed women who reside in communities with socio-economic and geographic isolations (such as rural settings or urban slums) may have limited opportunities for contraceptive uptake compared to individuals in settings with greater socio-economic advantage.

## Conclusion

In conclusion, this study established that uptake of modern contraceptives was higher among women who had exposure to IPV compared to those with no IPV exposure. Although the study cannot establish a cause-effect link or direction of causality between domestic violence and contraceptive use, findings of our study, building on evidence from previous research, suggest a bi-directional relationship between IPV and contraceptive use. We found that IPV tends to influence contraceptive use, while previous study reported that women’s use of contraception could lead to abuse and domestic violence ^46^ (Kaye *et al*). This is indicative of the complexity of the relationship between the two variables, and the present study provides empirical evidence that there is signiﬁcant community effect on IPV exposure and women’s contraceptive uptake. The study established that a significant proportion of the total variance in contraceptive use with respect to the different types of IPV is explained at the community level, thus suggesting the important roles of community contexts in the decision to use contraceptives among women in abusive union. Attention must therefore be given to the context-specific social and gender norms that affect women’s sexual and reproductive health in Nigeria.

## Strenghts and limitations of study

While the study has some strengths, including the use of a large nationally representative sample, and simultaneous analysis of both individual- and community-level variables; there are some drawbacks that must be considered while interpreting findings of the study. The use of primary sampling unit as a proxy variable for community may misclassify respondents into incorrect administrative boundaries. The use of secondary data constrains analysis to only the available variables collected in the survey, thus, relevant variables on community socio-cultural norms and other pertinent variables (such as prior experience of partner or non-partner abuse, duration or frequency of IPV) could not be explored. Further, the study did not explore the reverse impact of contraceptive use on IPV. Also, being a cross-sectional design, we can only infer association. Finally, the study was based on self-reported data which may have some element of social desirability bias particularly regarding the reporting of domestic violence.

## Data availability

The study used publicly available Demographic and Health Survey data, and is available on request on the website of DHS Program. To access the data, it is required to seek for permission from the DHS Program.
