# Supplementary material for: Community-level influences on women’s experience of intimate partner violence and modern contraceptive use in Nigeria: a multilevel analysis of nationally representative survey
Source: AAS Open Res. 2021 Nov 8;4:37. Originally published 2021 Jul 16. [Version 2] doi: 10.12688/aasopenres.13247.2 (PMC11077618; doi:10.12688/aasopenres.13247.2)
Supplement: Peer review history

# Reviewer report: Reviewer response for version 2

**DOI:** 10.21956/aasopenres.14451.r31031

**Published:** 2024-04-16

**Authors:** David Jean Simon

**Affiliations:** 1 Université Paris 1 Panthéon Sorbonne, Paris, France

This article deals with a very interesting topic. The relevance of this article is undeniable. Additionally, the article is well structured, the methods of analysis are relevant, and the tables are informative. However, before its approval, some changes need to be made.

 You will find my comments below.

1. I suggest the authors cite these recent articles. They will help them to refine their introduction and discussion.(Jean Simon D, et al., 2023 [Ref 1]), (Jamali Y, et al., 2024 [Ref 2]), (Jean Simon D, et al., 2024 [Ref 3]).
2.  I suggest that the authors provide more details in the first paragraph of the method section (see article above).
3. In the 'Statistical analysis' section, authors should mention why they used each type of analysis. For example, why simple frequency tables? Is it to decipher the profile of respondents? Why bivariate analysis? Why the model? Not all readers are familiar with these types of analysis.
4. What p-value was considered as a significant statistical level ?
5. The first paragraph in the results section should be the socio-demographic characteristics of the participants. The authors should present the profile of the respondents, which will make it easier to understand the results and the discussion.
6. Urban areas (20.8) (Add the percentage symbol behind 20.8)
7. The authors said ‘This suggests an increase in IPV prevalence as compared with previous study that established 23% IPV prevalence among married women from analysis of 2013 Nigeria DHS..’ This result is very interesting but what could explain it?
8. I suggest the authors take into account the women's profiles to refine the discussion.
9. There's a mistake in the title ‘Strengths and limitations of study’. Additionally, this section should be placed after the discussion.
10. Finally, Authors must add a section ‘Policy implication of findings’

Is the work clearly and accurately presented and does it cite the current literature?

Yes

If applicable, is the statistical analysis and its interpretation appropriate?

Yes

Are all the source data underlying the results available to ensure full reproducibility?

Yes

Is the study design appropriate and is the work technically sound?

Yes

Are the conclusions drawn adequately supported by the results?

Yes

Are sufficient details of methods and analysis provided to allow replication by others?

Yes

Reviewer Expertise:

Sexual and reproductive health, violence, criminology

I confirm that I have read this submission and believe that I have an appropriate level of expertise to confirm that it is of an acceptable scientific standard, however I have significant reservations, as outlined above.

## References

[1] : Prevalence and factors associated with condom use among sexually active young women in Haiti: evidence from the 2016/17 Haiti demographic and health survey. *BMC Womens Health* .2023;23(1) : 10.1186/s12905-023-02295-2 146 10.1186/s12905-023-02295-2 36991441 PMC10061838

[2] : Modern Contraception in Pakistan: A Cross-Sectional Study. *Population and Economics* .2024;8(1) : 10.3897/popecon.8.e106872 77-96 10.3897/popecon.8.e106872

[3] : Intimate partner violence among women in Togo: a generalised structural equation modeling approach. *BMJ Open* .2024;14(2) : 10.1136/bmjopen-2023-077273 e077273 10.1136/bmjopen-2023-077273 38373860 PMC10882340

# Reviewer report: Reviewer response for version 2

**DOI:** 10.21956/aasopenres.14451.r28980

**Published:** 2021-12-13

**Authors:** Winfred A. Avogo

**Affiliations:** 1 Department of Sociology and Anthropology, Illinois State University, Normal, IL, USA

The revised manuscript addressed my concerns well within the constrains of the dataset. I do not have any further comments.

Is the work clearly and accurately presented and does it cite the current literature?

Yes

If applicable, is the statistical analysis and its interpretation appropriate?

Yes

Are all the source data underlying the results available to ensure full reproducibility?

Yes

Is the study design appropriate and is the work technically sound?

Yes

Are the conclusions drawn adequately supported by the results?

Partly

Are sufficient details of methods and analysis provided to allow replication by others?

Yes

Reviewer Expertise:

Fertility and contraceptive use, social networks, social interaction and reproductive behavior, HIV/AIDS, migration and migrant health etc.

I confirm that I have read this submission and believe that I have an appropriate level of expertise to confirm that it is of an acceptable scientific standard.

# Reviewer report: Reviewer response for version 1

**DOI:** 10.21956/aasopenres.14369.r28819

**Published:** 2021-10-06

**Authors:** Fidelia A.A. Dake

**Affiliations:** 1 Regional Institute for Population Studies, University of Ghana, Accra, Ghana

The topic is interesting and the authors generally articulate the issues quite well. The intersection of IPV and modern contraceptive use in marriage has not been explored much. The manuscript is thus relevant. However, there are a number of issues that need to be addressed. Detailed comments are provided for each section.

**Introduction:**

- Paragraph 3, where papers are referenced include the names as well. For example, where it says "For instance 21, found that..." - It will be helpful to include the name(s) of the authors as well.
- Page 4 paragraph 2; The sentence, "Indeed, the assumption is that it ..." does not read well. The authors should reframe the sentence to make the meaning clearer. 

**Methods:**

- Variables: There is a time sequencing issue in the measurement of the dependent and independent variable. Ever experienced IPV could have happened anytime in the past while current use of modern contraceptives in the current union is in a different time. Basically, you cannot relate a previous experience of IPV to current use of modern contraception especially if current contraceptive use is in a union that is different from a union in which they experienced IPV. The sample should be limited to women in a marriage where they experience IPV and are using modern contraceptives. 
- IPV measure (B): the authors say the women who experience any two of the three questions asked but they appear to use all three questions. Please clarify.
- Other explanatory variables: Do they authors mean the average age at first marriage or just age at marriage? If a woman has been in more than one marriage and her current marriage is not her first marriage then which age at marriage is this study interested in? 
- Statistical analysis: Please clarify this; how many level two units were there in the multilevel model? 

**Results:**

 Multivariable analysis:

- Table 1: The chi squared vales and the p-values seem to have been interchanged. It does not make sense to have a p-value of 510.8156 or 169.0925 and a chi squared value of 0.000. Please check and correct this.
- Table 2: This table does not show the number of level 2 units. The table simply distributes the number of women by contextual classifications. For example what proportion of the women live in urban areas versus rural areas rather than the number of rural or urban units. For a multilevel model it is important to know and measure the level 2 units accurately.  
- Table 3 is confusing. The table headings seems to suggest that IPV is being regressed on IPV. The authors need to check this. Without this clarification, the whole of Table 3 is meaningless.
  Again, the dependent variable per the methodology outlined is modern contraceptive use coded as Yes "1" or No "1". The predictor variable in Table 3 is not clear. Please clarify this.
  The table can further be made more compact by taking the several rows of 1s representing the reference categories. Indicate the reference category for the explanatory variables like it was done with the IPV variables at the beginning of the Table. 
- One limitation of this study is that it does not explore community socio-cultural norms. It will be helpful to have a measure of these socio-cultural measures. For example patriarchy and polygamous unions etc. 
- Another limitation with the paper is that it fails to explore the reverse impact of CPR on IPV as alluded by the authors. This may be outside the scope of the current study but it is worth exploring. 
- The discussion and conclusions may need to be revised given the issues raised with the methods and the results.

Is the work clearly and accurately presented and does it cite the current literature?

Yes

If applicable, is the statistical analysis and its interpretation appropriate?

No

Are all the source data underlying the results available to ensure full reproducibility?

Yes

Is the study design appropriate and is the work technically sound?

No

Are the conclusions drawn adequately supported by the results?

Partly

Are sufficient details of methods and analysis provided to allow replication by others?

Yes

Reviewer Expertise:

Demography, Quantitative analysis

I confirm that I have read this submission and believe that I have an appropriate level of expertise to confirm that it is of an acceptable scientific standard, however I have significant reservations, as outlined above.

# Response

**Published:** 2021-10-27

**Authors:** Sunday Sunday

**Affiliations:** Federal University Oye-Ekiti, Nigeria

**Reviewer #2**

**Fidelia A.A. Dake**

**Comments:**

 The topic is interesting and the authors generally articulate the issues quite well. The intersection of IPV and modern contraceptive use in marriage has not been explored much. The manuscript is thus relevant. However, there are a number of issues that need to be addressed. Detailed comments are provided for each section.

**Response:**

 Thank you.

**Comments:**

**Introduction:**

 Paragraph 3, where papers are referenced include the names as well. For example, where it says "For instance 21, found that..." - It will be helpful to include the name(s) of the authors as well.

**Response:**

 Thank you for the comments. We have included the authors’ name (Pallitto & Campbell) as advised.

**Comments:**

 Page 4 paragraph 2; The sentence, "Indeed, the assumption is that it ..." does not read well. The authors should reframe the sentence to make the meaning clearer. 

**Response:**

 The sentence being referred to in paragraph 2 of page 4 has been properly rewritten as shown below:

Consequently, women are expected to submit to men’s authority and control on sexual matters. The culturally-laden social norms on intimate partner relationship reinforces women’s perception in the traditional societies that denying husbands sex is culturally and religiously unacceptable.

**Comments:**

**Methods:**

 Variables: There is a time sequencing issue in the measurement of the dependent and independent variable. Ever experienced IPV could have happened anytime in the past while current use of modern contraceptives in the current union is in a different time. Basically, you cannot relate a previous experience of IPV to current use of modern contraception especially if current contraceptive use is in a union that is different from a union in which they experienced IPV. The sample should be limited to women in a marriage where they experience IPV and are using modern contraceptives. 

**Response:**

 Thank you for the comments. The question on the experience of IPV specifically relates to the most recent partner/husband. The information on IPV was elicited from the respondents by asking the question: “ *Does (did) your (last) husband/partner ever do any of the following things to you?”. *This thus limits the sample only to women who experienced IPV in the recent union in which they used or didn’t not use modern contraceptives. This is clearly presented in the method section

**Comments:**

 IPV measure (B): the authors say the women who experience any two of the three questions asked but they appear to use all three questions. Please clarify.

**Response:**

 Thank you for the comments. We have corrected the phrase from ‘two questions’ to ‘three questions’.

**Comments:**

 Other explanatory variables: Do the authors mean the average age at first marriage or just age at marriage? If a woman has been in more than one marriage and her current marriage is not her first marriage then which age at marriage is this study interested in? 

**Response:**

 The study specifically controlled for age at first marriage. We have used same terminology for this variable throughout the manuscript. The study adjusted for age at first marriage because previous studies have established generally poor reproductive health outcomes for women who married early, notwithstanding the number of unions they have had.

**Comments:**

 Statistical analysis: Please clarify this; how many level two units were there in the multilevel model? 

**Response:**

 The level two of the multilevel analysis had 1,400 clusters (proxy for communities) across the country. This information has been included in the revised manuscript.

**Comments:**

**Results:**

 Multivariable analysis:

 Table 1: The chi squared vales and the p-values seem to have been interchanged. It does not make sense to have a p-value of 510.8156 or 169.0925 and a chi squared value of 0.000. Please check and correct this.

**Response:**

 Thank you for the comments. We have made the necessary corrections accordingly. All changes are tracked up.

**Comments:**

 Table 2: This table does not show the number of level 2 units. The table simply distributes the number of women by contextual classifications. For example what proportion of the women live in urban areas versus rural areas rather than the number of rural or urban units. For a multilevel model it is important to know and measure the level 2 units accurately.  

**Response:**

 Table 2 basically presents descriptive analysis of the contextual variables. For instance, the proportion of women living in rural areas (58.6%) was more than their counterparts in urban areas (41.4%). Essentially, the contextual variables were mainly generated by aggregating the level one (individual-level) variables and thereafter we took the proportion of women who had a particular attribute at the community level (level 2). Put differently, the level two variables were mainly generated by aggregating the individual-level variables at the level of primary sampling units (PSUs). We have provided this clarification in the revised manuscript.

**Comments:**

 Table 3 is confusing. The table headings seems to suggest that IPV is being regressed on IPV. The authors need to check this. Without this clarification, the whole of Table 3 is meaningless.

**Response:**

 Thank you for the comments. IPV is the explanatory variable and was regressed on modern contraceptive use (the outcome variable). We have 4 different sub-samples for the explanatory variable – (i) Physical IPV sub-sample (ii) Sexual IPV sub-sample (iii) Emotional IPV sub-sample (iv) Respondents with Any IPV. Different models focus on each of these 4 sub-samples. We have provided this clarity in the table and the prose.

**Comments:**

 Again, the dependent variable per the methodology outlined is modern contraceptive use coded as Yes "1" or No "1". The predictor variable in Table 3 is not clear. Please clarify this.

 The table can further be made more compact by taking the several rows of 1s representing the reference categories. Indicate the reference category for the explanatory variables like it was done with the IPV variables at the beginning of the Table. 

**Response:**

 As stated above, IPV is the explanatory variable and was regressed on modern contraceptive use (the outcome variable). We have 4 different sub-samples for the explanatory variable – (i) Physical IPV sub-sample (ii) Sexual IPV sub-sample (iii) Emotional IPV sub-sample (iv) Respondents with Any IPV. Different models focus on each of these 4 sub-samples. We have provided this clarity in the table and the prose. We have also made the table more compact by deleting rows of 1s representing the reference categories.

**Comments:**

 One limitation of this study is that it does not explore community socio-cultural norms. It will be helpful to have a measure of these socio-cultural measures. For example patriarchy and polygamous unions etc. 

 Another limitation with the paper is that it fails to explore the reverse impact of CPR on IPV as alluded by the authors. This may be outside the scope of the current study but it is worth exploring. 

**Response:**

 We acknowledge that lack of relevant variables on community socio-cultural norms and not exploring the reverse impact of CPR on IPV are study limitations. While the former was not available in the analysed secondary dataset, the latter issue is beyond the scope of the current study. These limitations have been acknowledged.

**Comments:**

 The discussion and conclusions may need to be revised given the issues raised with the methods and the results.

**Response:**

 The discussion and conclusion have been revised accordingly.

# Reviewer report: Reviewer response for version 1

**DOI:** 10.21956/aasopenres.14369.r28766

**Published:** 2021-08-13

**Authors:** Winfred A. Avogo

**Affiliations:** 1 Department of Sociology and Anthropology, Illinois State University, Normal, IL, USA

This study is on a topic of great concern to reproductive health of women in Sub-Saharan Africa. Contraceptive use is still low and Intimate Partner Violence (IPV) is high in the region and urgent measures are needed to reduce both to achieve Goal 5 of the Sustainable Development Goals on gender equality and empowerment of all women and girls.

 However, the main conclusion that modern contraceptive use was highest among married women who reported experience of IPV than those who did not was counter intuitive and more importantly, its explanation is not well sustained by the chosen theoretical and conceptual framework - theory of gender and power and neighborhood factors.

 As is explained in the manuscript, IPV is synonymous with women’s reproductive coercion – unprotected sex, control over women's  reproductive decision making, lack of negotiation skills etc. Perhaps one of the instances supporting contraceptive use among victims of IPV is when it is hidden from the male partner, but this study did not address the countervailing effects.

 Moreover, given this is a cross-sectional study rife with issues of temporality, I doubt how much credibility we should assign to this finding?

 There are also several confounder effects that probably should be adjusted for if the DHS data permits, some of these include: prior experience of partner or non-partner abuse, IPV duration or frequency – persistent of sporadic, parity, fertility intentions etc.

 The manuscript also references reverse causation - the possibility that use of contraceptives (covert or overt) may constitute a high risk of IPV - yet does not do more to test and rule out its effects.

 More could also be done to link the use of aggregate community variables to the theory or conceptual framework of the study - what about community level of education mediate the relationship between IPV and contraceptive use? How about community media access and community level of poverty?

Is the work clearly and accurately presented and does it cite the current literature?

Yes

If applicable, is the statistical analysis and its interpretation appropriate?

Yes

Are all the source data underlying the results available to ensure full reproducibility?

Yes

Is the study design appropriate and is the work technically sound?

Yes

Are the conclusions drawn adequately supported by the results?

Partly

Are sufficient details of methods and analysis provided to allow replication by others?

Yes

Reviewer Expertise:

Fertility and contraceptive use, social networks, social interaction and reproductive behavior, HIV/AIDS, migration and migrant health etc.

I confirm that I have read this submission and believe that I have an appropriate level of expertise to confirm that it is of an acceptable scientific standard, however I have significant reservations, as outlined above.

# Response

**Published:** 2021-10-27

**Authors:** Sunday Sunday

**Affiliations:** Federal University Oye-Ekiti, Nigeria

**Response to reviewers’ comments**

 Prof Winfred A. Avogo

**Comments:**

 This study is on a topic of great concern to reproductive health of women in Sub-Saharan Africa. Contraceptive use is still low and Intimate Partner Violence (IPV) is high in the region and urgent measures are needed to reduce both to achieve Goal 5 of the Sustainable Development Goals on gender equality and empowerment of all women and girls.

**Response:**

 Thank you.

**Comments:**

 However, the main conclusion that modern contraceptive use was highest among married women who reported experience of IPV than those who did not was counter intuitive and more importantly, its explanation is not well sustained by the chosen theoretical and conceptual framework - theory of gender and power and neighborhood factors.

 As is explained in the manuscript, IPV is synonymous with women’s reproductive coercion – unprotected sex, control over women's reproductive decision making, lack of negotiation skills etc. Perhaps one of the instances supporting contraceptive use among victims of IPV is when it is hidden from the male partner, but this study did not address the countervailing effects.

 Moreover, given this is a cross-sectional study rife with issues of temporality, I doubt how much credibility we should assign to this finding?

**Response:**

 Thank you for the comments. The finding that modern contraceptive use was highest among married women who reported experience of IPV did not come as a surprise. Previous studies in sub-Saharan Africa have established similar findings. Perhaps we did not provide sufficient evidence from literature on this, however, we have now done some revision and made additional inputs. We also did a due diligence to verify this result by undertaking a rigorous analysis. This finding has been well supported by the previous studies, and drawing on the literature, we have provided detailed explanation that women in abusive relationship possibly resort to covert contraceptive use due to their experience of IPV. Some relevant information included in the manuscript is provided below. Although the use of cross-sectional data poses some limitation, we have acknowledged these in the revised manuscript. Further, our contribution to the existing knowledge is mainly to examine if community-level characteristics modify the relationship between IPV and modern contraceptive use. We found that after incorporating community-level variables into the multilevel model, the relationship between IPV and contraceptive use became statistically insignificant. Some of the texts included in the revised manuscript are presented below:

Interestingly, we found that women who experienced different forms of IPV reported higher uptake of contraceptives than those who experienced no IPV. Previous studies have established similar findings . A study in Democratic Republic of the Congo that established a positive association between IPV and modern contraceptive use (Kidman et al, 2015). A multi-country study of 6 su b-Saharan African countries also established that women who experienced IPV had significantly higher odds of using contraceptives than those who had reported no exp erience of IPV (Alio et al, 2009) . This result has implication for women’s fertility intention and preference. P lausible explanation for high contraceptive use among women who experienced IPV is that occurrence of gender imbalances and exposures to the risk of violence in a marital dyad may force women to resort to a covert use of contraception [37, 38] in order to prevent having many children in an abusive union. Covert contraception (CC), which is the practice of using a family planning method without a partner’s knowledge, is a common practice among women in SSA [39] . Scholars have adduced that CC reflects the degree of how much autonomy a woman is able to exercise regarding her reproductive choices [40, 41] . Further , contraceptive use could trigger violence within a marital union that has palpable gender inequality. Given that women in some parts of Nigeria typically desire smaller families than their husbands/partners [42] , such desire may be accentuated by women’s exposure to spousal violence.

Further, we explored the roles of community-level factors on the relationship between IPV and contraceptive use. To the best of our knowledge, this is the first study to investigate this. Our findings provide some evidence that there are signiﬁcant neighbourhood effects on the relationship between IPV and modern contraceptive use. After incorporating community-level variables into the multilevel model, the relationship between two measures of IPV (physical IPV and emotional IPV) and modern contraceptive use became statistically insignificant.

**Comments:**

 There are also several confounder effects that probably should be adjusted for if the DHS data permits, some of these include: prior experience of partner or non-partner abuse, IPV duration or frequency – persistent of sporadic, parity, fertility intentions etc. The manuscript also references reverse causation - the possibility that use of contraceptives (covert or overt) may constitute a high risk of IPV - yet does not do more to test and rule out its effects.

**Response:**

 The variables suggested above are important for the study of this nature. However, these variables are not available in the dataset (except parity which has been included). We have acknowledged this limitation in the revised manuscript. The limitation of reverse causation has also been discussed in the revised manuscript.

**Comments:**

 More could also be done to link the use of aggregate community variables to the theory or conceptual framework of the study - what about community level of education mediate the relationship between IPV and contraceptive use? How about community media access and community level of poverty?

**Response:**

 As advised, we have revised the manuscript accordingly. The manuscript’s main contribution was to examine whether community-level characteristics modify the association between IPV and contraceptive use. The emerging findings have been discussed in the light of the literature and the theoretical framework for the study. All changes are tracked up.

**Comments:**

 Is the work clearly and accurately presented and does it cite the current literature?

 Yes

 Is the study design appropriate and is the work technically sound?

 Yes

 Are sufficient details of methods and analysis provided to allow replication by others?

 Yes

 If applicable, is the statistical analysis and its interpretation appropriate?

 Yes

 Are all the source data underlying the results available to ensure full reproducibility?

 Yes

 Are the conclusions drawn adequately supported by the results?

 Partly

**Response:**

 Thank you.
